# Intraoperative radiofrequency ablation for unresectable abdominal paraganglioma: a case report

**DOI:** 10.3389/fendo.2024.1346052

**Published:** 2024-04-15

**Authors:** Isabelle P. A. Magalhaes, Bibiana D. Boger, Nathalia L. Gomes, Guilherme L. P. Martins, Leomarques A. Bomfim, Gustavo F. C. Fagundes, Roberta S. Rocha, Fernando M. A. Coelho, Jose L. Chambo, Ana Claudia Latronico, Maria Candida B. V. Fragoso, Ana O. Hoff, Berenice B. Mendonca, Marcos R. Menezes, Madson Q. Almeida

**Affiliations:** ^1^ Adrenal Unit, Laboratory of Molecular and Cellular Endocrinology, LIM/25, Division of Endocrinology and Metabolism, Clinics Hospital, University of Sao Paulo Medical School, Sao Paulo, Brazil; ^2^ Division of Endocrinology, Santa Casa de Belo Horizonte, Belo Horizonte, Brazil; ^3^ Department of Internal Medicine, Federal University of Minas Gerais Medical School, Belo Horizonte, Brazil; ^4^ Interventional Radiology, Cancer Institute of São Paulo State (ICESP), University of Sao Paulo Medical School, Sao Paulo, Brazil; ^5^ Radiology Institute InRad, Clinics Hospital, University of Sao Paulo Medical School, Sao Paulo, Brazil; ^6^ Division of Urology, Clinics Hospital, University of Sao Paulo Medical School, Sao Paulo, Brazil; ^7^ Adrenal Unit, Laboratory of Hormones and Molecular Genetics LIM/42, Division of Endocrinology and Metabolism, Clinics Hospital, University of Sao Paulo Medical School, Sao Paulo, Brazil; ^8^ Division of Endocrine Oncology, Cancer Institute of São Paulo State (ICESP), University of Sao Paulo Medical School, Sao Paulo, Brazil

**Keywords:** paraganglioma, radiofrequency, ablation, intraoperative, unresectable

## Abstract

For pheochromocytoma and paraganglioma (PPGL), the efficacy of percutaneous ablative therapies in achieving control of metastatic tumors measuring <3 cm had been demonstrated in only few reports, and intraoperative radiofrequency ablation (RFA) of locally invasive primary PPGLs has not been reported. We presented the case of a 31-year-old man who had a 9-cm functioning unresectable PPGL. He was treated with 13 cycles of cytotoxic chemotherapy without objective tumor response, according to the Response Evaluation Criteria in Solid Tumors (RECIST). Subsequently, magnetic resonance imaging revealed a 9.0 × 8.6 × 6.0-cm retroperitoneal mass that extended to the inferior portion of the inferior vena cava, the inferior mesenteric artery, and the infrarenal aorta. Biochemical evaluation demonstrated high level of plasma normetanephrine (20.2 nmol/L, normal range <0.9 nmol/L). Genetic investigation showed the germline pathogenic variant c.1591delC (p. Ser198Alafs*22) in the *SDHB* gene. I^131^-metaiodobenzylguanidine scintigraphy was negative and Ga^68^-dotatate PET-CT scan showed high tumor uptake without distant metastases. On open laparotomy, tumor debulking was not possible. Therefore, intraoperative RFA was performed by a highly experienced team of interventional radiologists. At 12 months after the RFA, the tumor volume decreased from 208 to 45 mL (78%), plasma normetanephrine decreased from 20.2 to 2.6 nmol/L (87%), and the doxazosin dose was reduced from 16 to 8 mg/day. To our best knowledge, this was the first report on intraoperative RFA that markedly reduced the size of a large primary unresectable PPGL, along with clinical and biochemical responses.

## Introduction

Metastatic pheochromocytomas and paragangliomas (PPGLs) are defined by the presence of distant metastases at sites where chromaffin cells are physiologically absent (1). The latest World Health Organization (WHO) classification considered all PPGLs are considered to have metastatic potential, and replaced the previous term “malignant or benign” to metastatic or not metastatic (2). Approximately 10% to 15% of pheochromocytomas and a higher proportion (35% to 40%) of paragangliomas develop metastatic lesions (1). Large tumors >5 cm, paragangliomas (extra-adrenal location), multifocal disease, high plasma methoxityramine levels (normal range <0.1 nmol/L), normetanephrine level >5x the upper limit of normal reference range, and germline *SDHB* pathogenic variants had been associated with a higher risk for metastatic disease (3–6). Furthermore, patients with locally advanced pheochromocytoma (i.e., with capsular, vascular, and adipose tissue invasion) and/or positive locoregional lymph nodes butwithout evidence of distant secondary lesions at the time of diagnosis have an increased risk of recurrence (7).

Surgical removal is the main strategy to treat PPGLs (8, 9). The first choice treatment for patients with slow to moderate progression of metastatic and/or unresectable disease is radionuclide therapy with conventional or high-specific-activity ^131^I-metaiodobenzylguanidine (MIBG) or ^177^Lu-DOTATATE (10). Even in patients with metastatic disease, primary tumor resection appears to be associated with better blood pressure control and improved overall survival (11, 12). Radiation therapy and interventional radiology techniques can help control the symptoms of local metastases (especially bone lesions) to control local symptoms, and can also be effective for unresectable PPGL (13). However, there had been no studies that specifically addressed neoadjuvant MIBG or ^177^Lu-DOTATATE therapy for unresectable PPGL.

In a retrospective series, computed tomography (CT)-guided percutaneous ablative therapy by radiofrequency or cryoablation of 32 metastatic PPGLs, majority of metastatic lesions were in the bones and liver measuring up to 3 cm, were shown to effectively promote local control and palliate symptoms (14). However, intraoperative radiofrequency ablation (RFA) of large and unresectable primary PPGLs has not been previously demonstrated. Here, we reported the clinical, biochemical, and radiological outcome of an unresectable abdominal paraganglioma treated with intraoperative RFA.

## Case report

A 31-year-old male patient presented with symptoms of catecholamine excess, (such as sweating, weight loss, and poor control of hypertension), abdominal pain, and a retroperitoneal tumor. He was previously diagnosis of arterial hypertension at 18 years of age, but with no regular medical follow-up. Urinary normetanephrine level was 4260 ug/24h (normal range <732 µg/24h). Abdominal CT revealed a 10.5 x 7.2 x 9.5 cm retroperitoneal mass, which involved the inferior vena cava, inferior mesenteric artery, and infrarenal aorta. The patient underwent open laparotomy at another medical center, where an unresectable mass was revealed. Anatomopathological analysis confirmed the diagnosis of paraganglioma. Immunostaining was positive for chromogranin A, synaptophysin, GATA3 and Ki67 (5% to 10%). He was treated with 13 cycles of cytotoxic chemotherapy (cyclophosphamide, vincristine and dacarbazine) without objective tumor response, according to the Response Evaluation Criteria in Solid Tumors (RECIST) 1.1 criteria.

**Figure 1:**
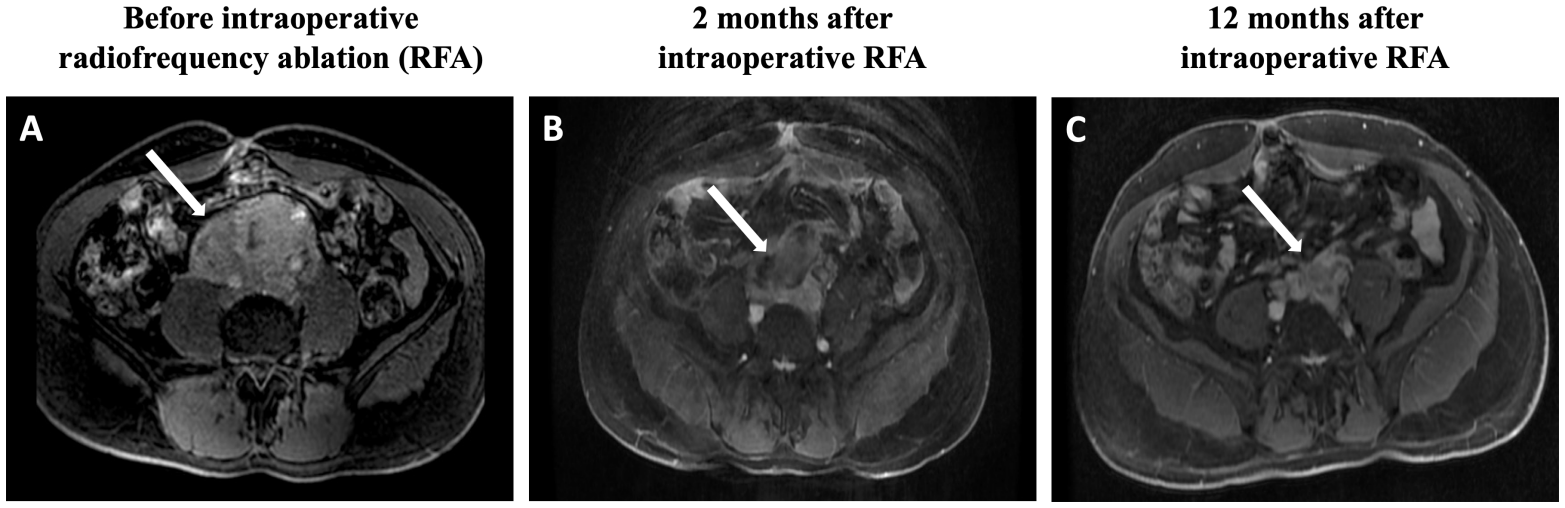
Magnetic resonance imaging findings: **(A)** Postgadolinium fat-saturation axial T1 weighted images reveal an extensive retroperitoneal mass, which measures 9.0 × 8.6 × 6.0 cm and involves the inferior portion of the vein inferior vena cava, inferior mesenteric artery, and infrarenal aorta. **(B)** The tumor size is reduced to 6.9 × 6.8 × 5.4 cm. **(C)** After 12 months of the RFA procedure, the tumor further shrinks to 7.2 × 4.8 × 4.1 cm. RFA, radiofrequency ablation.

The patient was then referred to our Institution for possible additional therapies. His blood pressure was well controlled with doxazosin 16 mg/day, propranolol 40 mg/day and losartan 100 mg/day. Magnetic resonance imaging (MRI) revealed an extensive retroperitoneal mass, which measured 9.0 x 8.6 x 6.0 cm (208 mL) and involved the inferior portion of the inferior vena cava, inferior mesenteric artery, and infrarenal aorta (**Figure 1A**). Biochemical evaluation demonstrated very high levels of plasma normetanephrine (20.2 nmol/L, normal range <0.9 nmol/L) and normal metanephrine level (0.2 nmol/L, <0.5 nmol/L). The cathecolamine metabolites were measured using liquid chromatography–tandem mass spectrometry.

**Figure 2:**
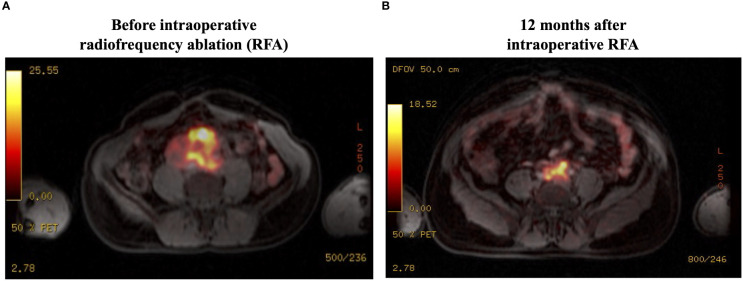
Ga^68^-DOTATATE PET-MRI findings: **(A)** There is high uptake (SUVmax, 32.5) in the abdominal mass, with local invasion but without distant metastatic disease. **(B)** There is a significant reduction in the tumor uptake (SUVmax, 18.5) at 12 months after RFA. SUVmax, maximum standardized uptake value; RFA, radiofrequency ablation.

Genetic investigation showed the germline pathogenic variant c.1591delC (p.Ser198Alafs*22) in the *SDHB* gene. I^131^-MIBG scintigraphy did not show any tumor uptake. The Ga^68^-DOTATE PET-MRI showed a high uptake, with a maximum standardized uptake value (SUV max) of 32.5 in the abdominal mass, with local invasion but without distant metastatic disease (**Figure 2A**). Because of the lack of alternative therapies, we proposed debulking by open laparotomy. Notably, the tumor did not have a clear cleavage plane with the large vessels and had a large caliber intratumoral vasculature. Therefore, intraoperative RFA was performed on the remaining lesion to promote tumor debulking. Intraoperative biopsy confirmed the diagnosis of paraganglioma with immunostaining positive for chromogranin and synaptophysin.

Intraoperative RFA was performed by a very experienced team, who used a Cool-tip™ RF E series ablation cluster electrode (Medtronic), which was 15.0-cm long and had a 2.5-cm active tip, under guidance by intraoperative ultrasound (GE healthcare LOGIQ™). Eight rounds were performed using an automatic protocol with maximum output power of 200 W lasting 6-12 min each, according to the behavior/impedance which ranged from 80 to 110 ohms during the procedure, in order to contemplate the middle and anterior portions of the lesion, reaching approximately 80% of the tumor volume. The left posterior portions of the lesion were only partially ablated because of their proximity to the left ureter. The oral anti-hypertensive medications (doxazosin, propranolol and losartan) were discontinued on the day of the procedure. During the procedure, invasive hemodynamic monitoring was performed by invasive arterial and central venous pressure monitoring. The patient’s blood pressure was controlled intra-operatively with continuous intravenous sodium nitroprusside. There were no reported immediate complications in the intra- and postoperative period. After the procedure, no active bleeding was observed, and there was a partial reduction in the tumor volume associated with areas of coagulative necrosis/charring in the periphery.

After 2 months of the RFA procedure, the dose of doxazosin was decreased from 16 to 8 mg (50% reduction), the plasma normetanephrine level dropped to 4.6 nmol/L (79% reduction), and the tumor size decreased to 6.9 x 6.8 x 5.4 cm (88.8 mL, 57.3% reduction in volume) on MRI (**Figure 1B**). After 12 months, the tumor further shrunk to 7.2 x 4.8 x 4.1 cm (45 mL, 78.4% reduction in volume), and plasma normetanephrine decreased to 2.9 nmol/L (86% reduction) (**Figure 1C**). Ga^68^-dotatate PET-MRI showed a significant reduction in the SUVmax from 32.5 to 18.5 after 12 months of the RFA (**Figure 2B**).

## Discussion

Compared with other solid tumors, metastatic and/or unresectable PPGLs are more indolent and have an estimated 5-year survival rate of 34% to 74% (15). Approximately 35% of the metastatic PPGLs have synchronous metastases upon initial diagnosis (10). The most frequent sites of metastases are the bone and lymph nodes (2). Lung and liver metastases are associated with poor outcome (15). The approach to systemic disease is mainly palliative and divided by two main scenarios: rapid progression vs. slow or moderate progression. Rapid progression is defined by a high tumor burden for <6 months, especially if there are secondary lesions in the liver and lung. The first choice treatment for cases of slow to moderate progression is radionuclide therapy (13). In fact, treatment with ^131^I-MIBG (dose ranging from 492 to 1,160 mCi) promote a 22% partial objective response according to the RECIST 1.1 (16). However, 35% of patients showed disease progression after 1 year. A prospective trial showed that high-specific-activity ^131^I-MIBG (500 mCi twice at 3 to 6 month intervals) was associated with a 30% partial response rate and 68% stable disease rate after a second dose, based on RECIST (17). Furthermore, a 50% reduction in all antihypertensive drugs, lasting at least 6 months, occured in 25% of the patients.

In the case reported here, the paraganglioma did not show any uptake on I^131^- MIBG scintigraphy. The patient had no objective tumor response after 13 cycles of cytotoxic chemotherapy (cyclophosphamide, vincristine and dacarbazine). Indeed, stable disease had been the most frequent outcome of metastatic PPGLs after the available systemic therapies (13). Because the Ga^68^-DOTATE PET-MRI showed a positive uptake, ^177^Lu-DOTATATE would have been a treatment option, but this radionuclide therapy is not available in the public health system in Brazil. The largest study to date reported an overall response rate of 23% after ^177^Lu-DOTATATE in 30 patients with metastatic PPGLs (18). Moreover, a metanalysis showed that treatment with ^90^Yor ^177^Lu‐based peptide receptor radionuclide therapy achieved an objective response rate of 25% and a disease control rate of 84%. Clinical and biochemical responses were seen in 61% and 64% of the patients, respectively (19).

For this present case, a debulking open laparotomy was proposed by a highly experienced surgeon because of the lack of alternative therapies, but it was not anatomically feasible. The main indication for RFA was the presence of large vessel invasion (inferior vena cava, inferior mesenteric artery, and infrarenal aorta). Therefore, intraoperative RFA was performed and led to significant objective tumor and biochemical responses. Percutaneous RFA has been used for primary pheochromocytomas (non-invasive and non-metastatic) in patients who are poor candidates for surgery (20–22). Although percutaneous ablative therapies can be used for the local management of small metastatic lesions, to the best of our knowledge, intraoperative RFA of an unresectable PPGL has not been previously reported.

Both intraoperative and percutaneous RFA have been increasingly applied in the management of unresectable pancreatic cancer, with a high clinical success rate (23). In addition, intraoperative or percutaneous RFA was shown to be safe, well-tolerated, and effective treatment in achieving destruction in patients with unresectable primary and metastatic hepatic malignancies (22). Curley et al. (22) used RFA to treat 169 tumors (median diameter 3.4 cm, range 0.5 to 12 cm) in 123 patients with primary liver cancer (39%) or metastatic liver lesions (61%). After a median follow-up of 15 months, tumor has recurred in only 3 of 169 treated lesions (1.8%). The efficacy and safety of CT-guided ablative therapy for metastatic PPGLs has been demonstrated in small series (24, 25). McBride et al. (25) evaluated the efficacy of percutaneous ablation in 10 patients with metastatic PPGLs. Most of the lesions were located in the liver and bones with mean tumor size of 2.1 cm (range, 0.5 to 4.6 cm). Successful ablation without evidence of recurrence was achieved in 56% (15 of 27) of the primarily treated lesions in patients who had available follow-up imaging (25). In 2019, Kohlenberg et al. (14) expanded this series from Mayo Clinic and studied 123 lesions from 32 patients with metastatic PPGL were treated with percutaneous CT-guided RFA or cryoablation. Radiological local control was achieved in 86% of the lesions. Clinical improvement (pain or symptoms of catecholamine excess) was achieved in 12 of 13 (92%) procedures, with need for intravenous blood pressure medications in 14% of the procedures and procedure-related death in only 1 patient (14).

RFA using microwave ablation for larger tumors may represent a very effective treatment for large tumors. Microwave ablation offers advantages such as faster treatment times, larger ablation zones, no heat sink effect in tumors close to large vessels and potentially improves success rates. Further research and clinical studies are warranted to explore this approach.

In conclusion, we reported the first intraoperative RFA for a large primary unresectable PPGL, with marked clinical, biochemical, and tumor responses. Blood pressure was safely controlled with intravenous medication during the procedure. The patient did not have any serious complications after RFA. Therefore, we propose this novel, effective, and safe approach for debulking a large and unresectable primary PPGL.

## Data availability statement

The raw data supporting the conclusions of this article will be made available by the authors, without undue reservation.

## Ethics statement

Written informed consent was obtained from the patient to perform genetic analysis and for the publication of any potentially identifiable images or data included in this article.

## Author contributions

IM: Data curation, Formal analysis, Investigation, Validation, Visualization, Writing – original draft. BB: Data curation, Formal analysis, Investigation, Validation, Visualization, Writing – original draft. NG: Data curation, Validation, Writing – review & editing. GM: Writing – review & editing, Data curation, Formal analysis, Investigation, Methodology, Software. LB: Investigation, Methodology, Software, Writing – review & editing. GF: Formal analysis, Investigation, Supervision, Writing – review & editing. RR: Formal analysis, Investigation, Supervision, Writing – review & editing. FC: Supervision, Writing – review & editing, Investigation, Software. JC: Investigation, Supervision, Writing – review & editing. AL: Conceptualization, Supervision, Validation, Writing – review & editing. MF: Supervision, Validation, Visualization, Writing – review & editing. AH: Conceptualization, Supervision, Validation, Writing – review & editing. BM: Conceptualization, Formal analysis, Supervision, Writing – review & editing. MM: Data curation, Formal analysis, Investigation, Methodology, Software, Supervision, Writing – review & editing. MA: Conceptualization, Supervision, Writing – review & editing.
